# An automatic service for the personalization of ventricular cardiac meshes

**DOI:** 10.1098/rsif.2013.1023

**Published:** 2014-02-06

**Authors:** Pablo Lamata, Matthew Sinclair, Eric Kerfoot, Angela Lee, Andrew Crozier, Bojan Blazevic, Sander Land, Adam J. Lewandowski, David Barber, Steve Niederer, Nic Smith

**Affiliations:** 1 Department of Biomedical Engineering, King's College of London, St Thomas' Hospital, London SE1 7EH, UK; 2 Department of Computer Science, University of Oxford, Oxford OX1 3QD, UK; 3 Division of Cardiovascular Medicine, Radcliffe Department of Medicine, University of Oxford, Oxford OX3 9DU, UK; 4 Department of Cardiovascular Science, University of Sheffield, Royal Hallamshire Hospital, Sheffield S10 2JF, UK

**Keywords:** computational physiology, cardiac modelling, computational mesh

## Abstract

Computational cardiac physiology has great potential to improve the management of cardiovascular diseases. One of the main bottlenecks in this field is the customization of the computational model to the anatomical and physiological status of the patient. We present a fully automatic service for the geometrical personalization of cardiac ventricular meshes with high-order interpolation from segmented images. The method is versatile (able to work with different species and disease conditions) and robust (fully automatic results fulfilling accuracy and quality requirements in 87% of 255 cases). Results also illustrate the capability to minimize the impact of segmentation errors, to overcome the sparse resolution of dynamic studies and to remove the sometimes unnecessary anatomical detail of papillary and trabecular structures. The smooth meshes produced can be used to simulate cardiac function, and in particular mechanics, or can be used as diagnostic descriptors of anatomical shape by cardiologists. This fully automatic service is deployed in a cloud infrastructure, and has been made available and accessible to the scientific community.

## 1. Introduction

The field of computational physiology has progressively accelerated over the last four decades. Its importance is particularly significant for modelling the electromechanical function of the heart [1,2], as an aid to clinicians in stratifying disease through novel indices [3], in the design of mechanical reinforcement of infarct regions [4], in predicting cardiac outcomes [5] and also in patient selection for procedures such as cardiac resynchronization therapy [5–8]. However, the full clinical translation of these modelling techniques still requires remaining challenges to be addressed. In particular, these include determining the appropriate computational complexity of the biophysical models and the need for model personalization. Customization of computational models involves two main aspects: to capture the patient-specific anatomy of interest and to estimate the parameters of the constitutive biophysical laws that govern the equations of the model. This article focuses on the geometrical aspect of personalization and on specific high-order interpolation meshes used for mechanical simulations.

Computational meshes define a geometric representation of anatomy, providing the domain for solving the mathematical description of physiological processes. They are required to be both geometrically accurate and to provide good numerical stability when used to solve governing equations. For many physiological simulations, the computational domains are described using unstructured linearly interpolated tetrahedral meshes. This is primarily owing to the availability of tools for automatic meshing of complex geometries, such as *TetGen* (http://tetgen.org) or *Tarantula* [9], their relative simplicity and their well-characterized properties [10]. Nevertheless, linear interpolation schemes in tetrahedral elements have convergence disadvantages and can introduce significant numerical errors in calculating solutions for the important class of incompressible soft tissue deformation simulations [11]. An alternative solution is the use of meshes with high-order interpolation, such as cubic Hermite and cubic Lagrange meshes. These schemes provide an efficient representation of the mechanical state of an organ as well as improved accuracy [12]. They also enable a much more compact and geometrically smooth and continuous representation, and thus a reduction of the computational cost in simulations. For these reasons, high-order interpolation meshes are a popular choice for the simulation of cardiac mechanics [3,4,6,8].

There are two broad approaches for the personalization of geometrical meshes: the direct construction of a mesh from segmented images [13,14] and the customization of a mesh from an existing mesh model [13,15]. Whereas the literature for linear meshes is extensive [10], the translation of these techniques to meshes with higher order of interpolation remains to be fully developed. Recent advances have solved the integration of image registration methods by a variational warping technique [15], have developed a topological solution to represent the four-chamber cardiac anatomy [16] and have proposed extraordinary vertices in order to represent atrial chambers [14]. However, despite these efforts, no robust and automated tools are currently available for the personalization of ventricular meshes with high-order interpolation required for clinical application or large population analyses. Furthermore, while stability and convergence of mechanical simulations are dependent on the regularity and quality of mesh elements, there are currently no mesh generation tools that seek to guarantee these properties.

This article presents a versatile and fully automatic meshing solution for cubic Hermite and cubic Lagrange cardiac ventricular meshes maximizing both anatomical accuracy and simulation stability. The two main methodological contributions are a robust strategy of template synthesis and alignment in order to maximize image registration convergence, and a mesh postprocessing step in order to improve mesh quality. The method is thoroughly tested with a wide set of cases, evaluating its versatility, accuracy, quality, robustness and computational time. All resulting meshes are published, and the proposed solution is deployed as a web service using cloud technology, enabling easy access and use by the scientific community.

## 2. Material and methods

A binary mask describing the domain of the ventricular anatomy is the only data required for the mesh personalization, which is performed with the combination of four main steps: (i) the analysis of the myocardial shape in order to tailor a template mesh, (ii) a fast and robust binary image registration, (iii) a variational technique for mesh warping and (iv) a postprocessing step to improve mesh quality. The result is a cubic interpolation mesh (in *EX* format, see *FieldML* standard [17] and *cmGui* [18] as a tool to visualize and convert) fitted to the domain described by the binary mask.

**Figure 1.**
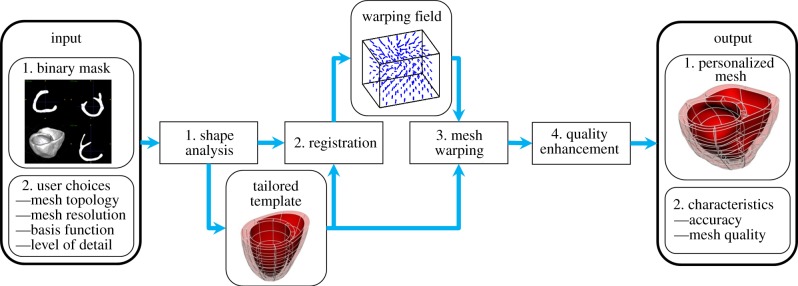
Overview of the mesh customization service. User provides the definition of myocardial domain by a binary image (from any modality), and a set of choices depending on his/her specific requirements. The service then performs: (1) a shape analysis to customize an idealized template; (2) an image registration to assess the deformation field between template and provided anatomy; (3) a variational mesh warping to deform the high-order interpolation mesh and (4) a postprocessing linearizing step to enhance the quality of the mesh. (Online version in colour.)

In the process, the user can choose topology, basis functions and resolution of the computational mesh, and the balance between quality and the level of detail (LoD) in which to capture the anatomy. An overview of the methodology is presented in figure 1, and the description of its main functional blocks is provided below.

### 2.1. Shape analysis for a tailored template mesh

**Figure 2.**
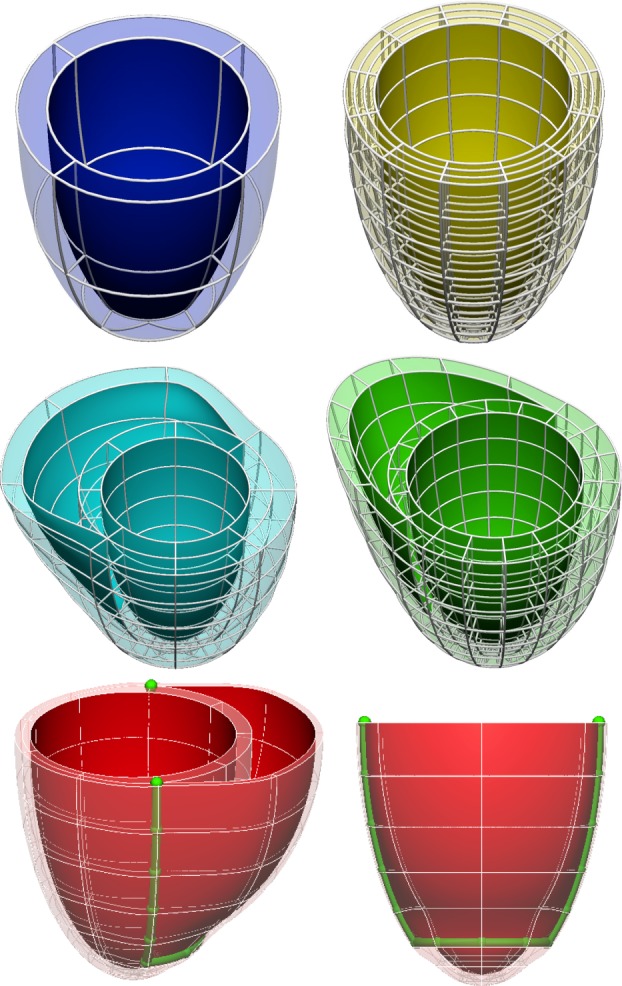
Examples of template meshes of the LV and the BiV anatomy with different resolutions and dimensions of the right ventricular blood pool. Bottom row highlights in green the squared insertion line of the RV into the LV (line that joins the ventricular cusps referred in [19]).

Ventricular anatomy is synthesized with truncated ellipsoids, with user-defined topology and number of elements in each local material direction, as illustrated in figure 2. Valve planes are thus not represented by this topology. Meshes are structured and have local material coordinates aligned with the main directions of the left ventricular anatomy (circumferential, longitudinal and radial), and collapsed elements in the apex.

The anatomical variability of the biventricular (BiV) shape in humans and other species makes the use of a single template mesh problematic for embedding within a robust meshing solution. The strategy adopted here is thus to tailor the template mesh to a set of measurements from the binary mask used as input (another approach is the choice of the most similar reference from a database [20]). This significantly increases the rate of convergence and accuracy of the subsequent image registration, and the quality of the resulting mesh, as the initial regular template is much closer to the final solution.

**Figure 3.**
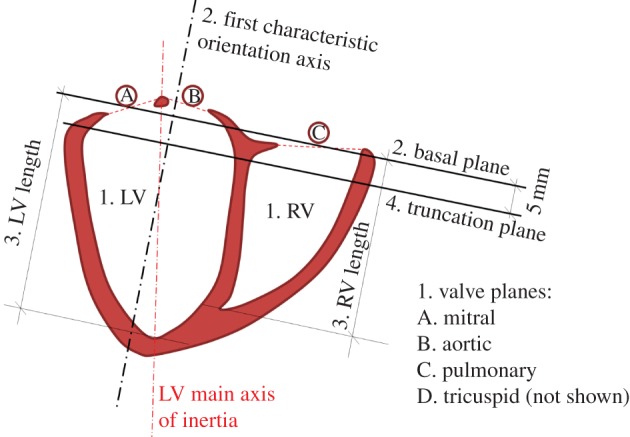
Shape analysis of a BiV binary mask, with numbers illustrating its sequential steps. Note how the main axis of inertia of the LV is not aligned with the perpendicular to the basal plane. (Online version in colour.)

The geometrical features extracted from the input image are wall thickness and relative size of the right ventricle (RV) to the left ventricle (LV). The analysis is done with a combination of steps (figure 3): (i) identification of the blood pools and valve planes from the image, (ii) estimation of the spatial orientation of the heart, (iii) computation of geometrical dimensions of RV and LV and (iv) an optional truncation of the basal anatomy.

Blood pools and valve planes are identified in the binary mask by computing the convex hull of the shape and finding the two main cavities and openings in the shape through a combination of morphological (erosion) operations. Characteristic axes of the cardiac anatomy are used to define spatial orientation. The main vertical axis is defined perpendicular to the basal plane, which is defined as the plane that minimizes the distances to voxels labelled as part of the valves in the previous step.

The second left-to-right axis is computed as the projection in the basal plane of the vector joining the centre of mass of LV and RV. The spatial orientation of the heart is completed by the cross product of these two main axes. In the case of datasets with a large distance between slices, for example short axis cine-MRI acquisition, the first axis is defined by the normal to the image slice plane. The variance of the points in each of these three directions leads to the definition of an anisotropic scaling factor.

The size of the RV and LV is computed by the projection of the shape of the blood cavities into the characteristic axes of spatial orientation. The variance of the coordinates in the direction of characteristic axes defines the size of the ellipsoidal shapes used to synthesize the RV and LV. The length of the RV defines the insertion level where the apex of the RV attaches to the LV. Finally, the optional truncation of basal anatomy is defined at 5 mm below the basal plane.

### 2.2. Image registration and *level of detail*

The warping function between the shape of the patient's anatomy and a synthesized template is obtained by image registration. This meshing service uses ShIRT, the Sheffield Image Registration Toolkit, a robust and efficient registration engine. The initial alignment between images is set by the affine transformation defined by the characteristic orientation axes and scaling factors extracted from the input image (see previous section).

The details of the underlying image registration algorithm used in this paper and examples of its use are presented in [21]. The fitting accuracy and quality (numerical stability) of the resulting mesh strongly depend on this registration step. These two qualities are in typically in tension [22], and thus a parameter named LoD is provided to the user to balance these two important characteristics. LoD ranges from 1, coarse and most stable, to 5, accurate but with lowest chances of stability. The internal parameters that control this choice are the number of registration passes and the node spacing, the distance between each pair of adjacent control nodes that define the set of degrees of freedom in the registration process (see [21] for further details about this registration parameter). Thus, LoD 1 to 5 has 1 to 5 registration passes, respectively, and the node spacing in each pass decreases from 10 to two voxels, in steps of two voxels.

In many clinical situations, only a few short or long axis slices are available, most commonly in dynamic studies. The solution adopted for these cases exploits the versatility of ShIRT to specify the image domain where the similarity metric is computed. A nearly isotropic resolution image from the original data is built, and the registration is only guided by the slices that have original data (there is thus no need to interpolate these data, the new voxel locations are filled with null intensity values).

### 2.3. Mesh warping

Once the warping function between the template and patient's anatomy has been calculated by image registration, the template mesh is warped using an accurate variational technique. The key in this step is that a mesh is deformed, not by an independent and local warping of each of its nodal degrees of freedom, but by warping the whole domain that these degrees of freedom represent, the whole continuum. This technique calculates the projection of the deformation field into the degrees of freedom of the computational mesh by minimizing the L2 norm of the projection error. The reader is referred to [15] for a detailed explanation of this variational technique, together with a comparison to a nodal warping technique.

### 2.4. Quality enhancement

Mesh quality is a metric of the geometrical regularity of its constituent elements (the more skewed and warped the element, the lower its quality), and it is an index that predicts the stability of simulations [22]. Stability refers to the numerical convergence of the mechanical solver to a solution.

The structured cubic meshes generated to represent the ventricular anatomy have a subset of internal nodal degrees of freedom that do not modify the epicardial and endocardial surfaces that capture the anatomy. The objective of this postprocessing step in the pipeline is thus to modify these specific degrees of freedom in order to improve the regularity, the quality of the mesh, with no loss of accuracy. Specifically, this is implemented as a linearization of the element in the transmural direction. The nodal derivatives in the radial direction are modified to point in a straight line between opposite nodes across the wall.

### 2.5. Deployment of meshing service: heartgen

A web service called heartgen allows users to upload segmentation data manually through a form and receive results via email or to submit data programmatically and receive results directly. Heartgen is hosted as part of the Anatomical Model Database (http://amdb.isd.kcl.ac.uk) [23,24], a database of geometric and functional model data. Heartgen is also available as a service within the infrastructure of the VPH-Share project (http://www.vph-share.eu/), an initiative to integrate and offer computational tools in order to apply, and develop, analysis and simulation workflows. The tool itself is implemented in MATLAB in conjunction with *ShIRT* [21], used to register images, and *cmGui* [18], a data visualization tool used to generate binary images from meshes and captions of the results. MATLAB is used to warp the template mesh to the input binary data and to orchestrate the workflow. The reader is referred to the study of Kerfoot *et al*. [23] for full details about the deployment of a preliminary pipeline of heartgen.

## 3. Results: performance analysis

### 3.1. Versatility

**Figure 4.**
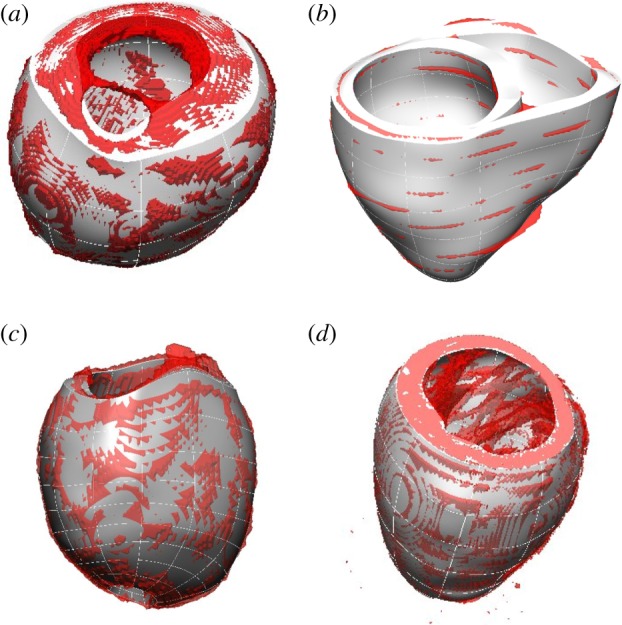
Versatility of the meshing solution, illustrated with four examples of meshes automatically personalized. (*a*) LV of a dilated cardiomyopathy patient from SSFP MRI; (*b*) BiV truncated mesh from a sparse cine-MRI dataset; (*c*) LV with a hole in the apex from SSFP MRI, needed for the simulation of an assisted device [25] and (*d*) LV of a mouse with detailed papillary and trabecular anatomy smoothed by the mesh. Images show the mesh (white smooth geometry) overlapping with the isosurface of the binary mask (red geometry) provided as input.

The method has been successfully applied to cardiac geometries with different pathologies at human scale (dilated or normal hearts), different species (human or animal) and different amounts of anatomical information (from a sparse dataset of 6/8 slices, to a high-resolution animal experimental dataset with 29 μm isotropic voxel size and 222 × 233 × 252 voxels; see figure 4). The anatomical detail of trabecular anatomy is smoothed by the choice of a low number of elements and a high-order interpolation.

### 3.2. A compromise in the *level of detail*

Personalization of a computational mesh is limited by the compromise between geometrical accuracy, the level of anatomical detail captured and mesh quality, a characteristic that predicts simulation stability. For a detailed analysis of this compromise in the simulation of cardiac mechanics, the reader is referred to Lamata *et al*. [22]. This section reports the effect of the selection of the LoD parameter ruling the registration process in the meshing service. Two cases with the average shape of the human BiV anatomy (A1, A2) are selected from two statistical atlases used for segmentation, [26] and [27], respectively.

Mesh quality (*Q*) is characterized by the Jacobian ratio, previously identified to be the quality metric with the best compromise between performance versus computational cost in the prediction of simulation stability [22]. This metric analyses the transformation from local to global coordinates, measuring the homogeneity of each volume differential. The determinant of the Jacobian matrix of this transformation is the volume differential, and thus3.1
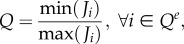
where *Q^e^* represents the discrete set of quadrature points (*i* is each of these points) within an elemental volume.

**Figure 5.**
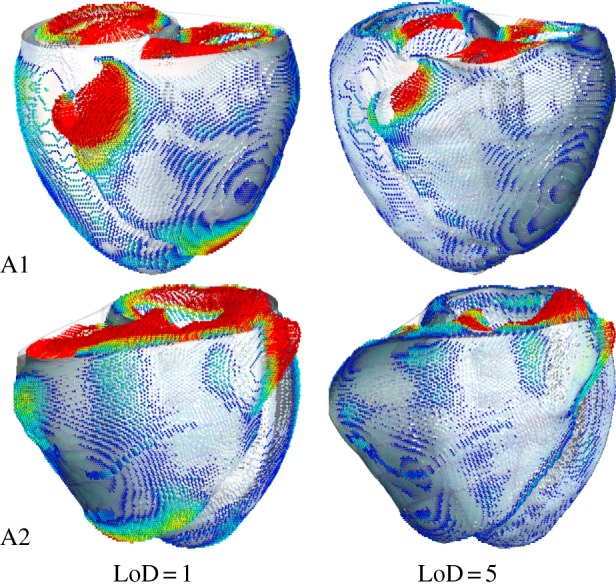
Personalization results from the two average anatomies (A1, A2) with the two extreme values of LoD, the parameter that controls the compromise between accuracy and stability of the resulting mesh. Images represent the final mesh (white surface) with the fitting error colour coded from blue (error = 0 mm) to red (error = 1 mm).

Fitting accuracy is evaluated by the error between the segmented image and the mesh (see illustration of this metric in figure 5). This error is defined in equation (3.2) as the Euclidean distance, expressed in millimetres, from the nodes of the isosurface *N*_iso_ of the binary image to the closest point of the external surface of the cubic mesh CP_mesh_. Error was integrated through the surface of the mesh (*S*) using Gaussian quadrature as described in [15]3.2
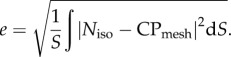

**Figure 6.**
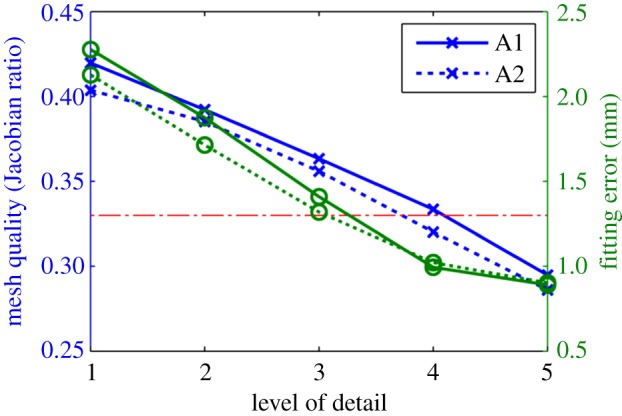
Impact of the choice of the LoD in geometrical accuracy (lines with crosses), characterized by the average fitting error (see equation (3.1)), and mesh quality (lines with circles), characterized by the Jacobian ratio (see equation (3.2)), in the two average anatomies (A1, A2). Horizontal dashed line is the empirical threshold of mesh quality [22]. (Online version in colour.)

Results of quality and accuracy are reported in figure 6. The main discrepancy between the binary segmentations and the final meshes is owing to the topology of the template, which has a unique opening at the base of each ventricle, and not two representing the valve planes. Regions of biggest fitting error are thus at this basal site (figure 5). Note that the fitting error results reported in figure 6 will be significantly lower if the binary segmentation is cropped at the base to remove the valve planes, see an example in figure 4*b*. The apex of the RV is the other region with the biggest errors when using small values for the LoD.

Mesh quality values are low compared with standard thresholds of the Jacobian ratio in the literature owing to the presence of collapsed elements in the apex of the LV. An empirical threshold of this quality metric for the cardiac BiV anatomy is 0.33 (horizontal dashed-dotted line in figure 6), with sensitivity of 78% and specificity of 58% for the simulation of a heartbeat [22].

### 3.3. Improving quality without accuracy loss

**Figure 7.**
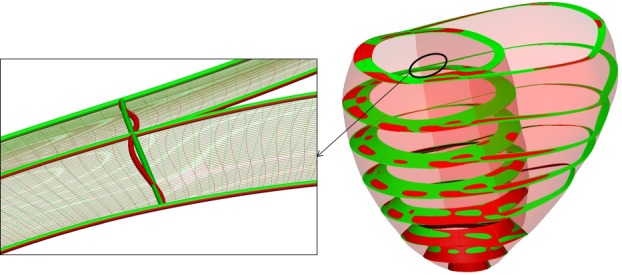
Quality mesh enhancement in the meshing pipeline (step 4 in figure 1): comparison of the output of the warping step (red) and improved mesh (green) for case A1 with LoD = 3.

**Figure 8.**
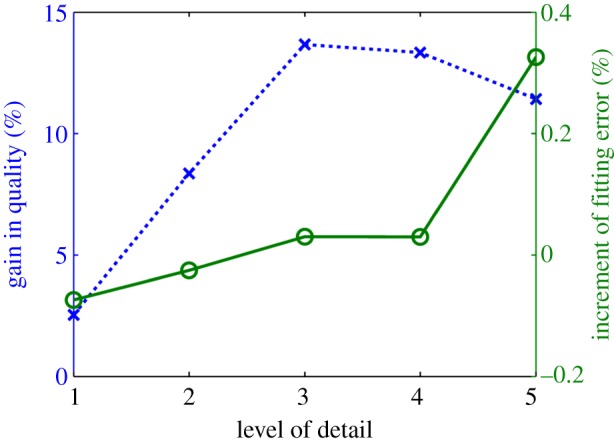
Impact of the mesh enhancement in the meshing pipeline (step 4 in figure 1) for each LoD. Values represent the mean results in the two average anatomies (A1, A2), quality in dashed line, and fitting error in solid line. (Online version in colour.)

Meshes of the previous section with the two average anatomies (A1, A2) are generated again, this time without linearization of the transmural degrees of freedom, the process illustrated in figure 7. Comparison results are reported in figure 8. Average accuracy for all LoD is decreased by a nominal amount (0.006 mm) owing to the flattening of the basal external surface. Average mesh quality is improved by 8.7% (an increment of 0.028 of the average Jacobian ratio).

### 3.4. Performance with sparse datasets

**Figure 9.**
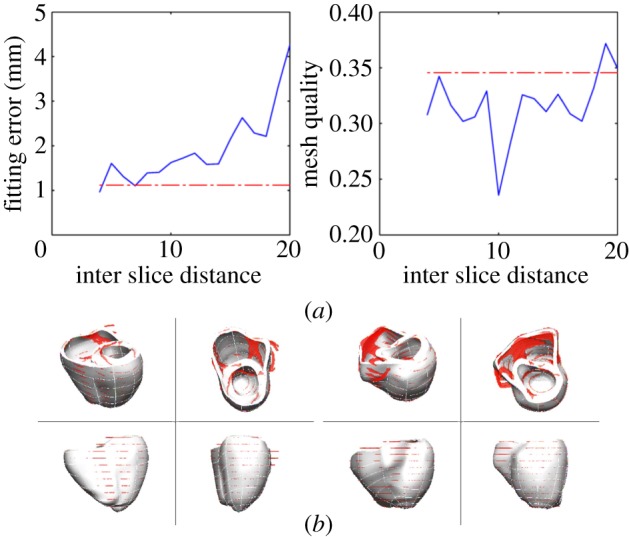
Performance with sparse datasets generated with increased interslice distance from the two average anatomies (A1, A2): (*a*) accuracy and mesh quality from a stack of short axis slices (solid line) compared with the result from full isotropic 1 × 1 × 1 mm resolution (dashed line). (*b*) Result with an interslice distance of 9 mm in the two average anatomies (red isosurface is the anatomical data, and white surface is the resulting mesh).

The performance of the meshing service with sparse datasets (i.e. with a large distance between image slices) is tested in a set of cases generated from the two average anatomies (A1, A2). The anatomy from 1 × 1 × 1 mm isotropic resolution is oriented in the direction of the LV axis of inertia, and a stack of short axis slices is generated, with increasing interslice distance, from 4 to 20 mm. Meshes are built with an LoD 3, and degradation in terms of accuracy and quality is characterized by the average performance of these two cases. Geometrical fitting error is evaluated against the original image with isotropic resolution. Results illustrate the gradual degradation of performance in terms of fitting error with decreasing amounts of anatomical information (figure 9).

### 3.5. Robustness

Robustness of the meshing service is tested in a set of 216 and 39 cases of the left and BiV anatomy, respectively. LV cases are manually segmented from short axis stacks of 7/8 slices at end diastole from dynamic MRI studies (the reader is referred to [28] for a full description of this cohort). BiV cases are automatically segmented with methods described in [20] from whole-heart MRI images captured by a balanced steady-state free precession (b-SSFP) protocol, with average dimensions of 153 × 363 × 396 voxels and average spacing of 1.278, 0.850, 0.850 mm. Binary masks of BiV cases are cropped at the base in order to remove the valve planes and represent the same topology of the template mesh. LoD 1 and 2 are chosen for the LV and BiV cases, respectively. Mesh resolution is 1, 12, 6 and 2, 9, 8 elements in radial, circumferential and longitudinal directions for LV and BiV cases, respectively.

**Figure 10.**
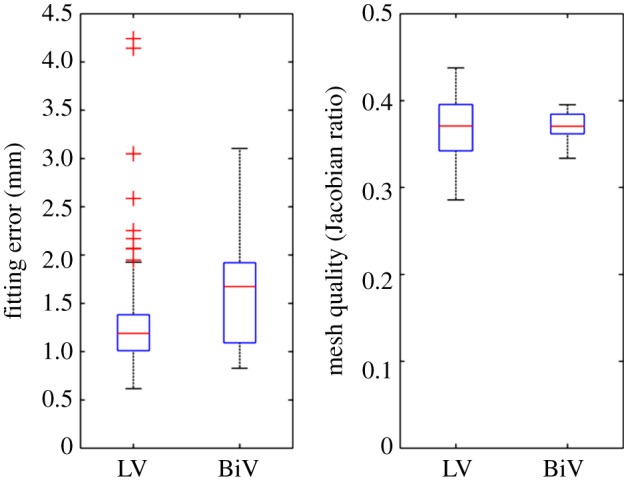
Box plot of the meshing accuracy (characterized by the fitting error) and quality results on the cohorts of 216 left ventricular (LV) and 39 BiV cases. Outliers in fitting errors in the LV cohort are owing to slice shifts (see an example in figure 11). (Online version in colour.)

**Figure 11.**
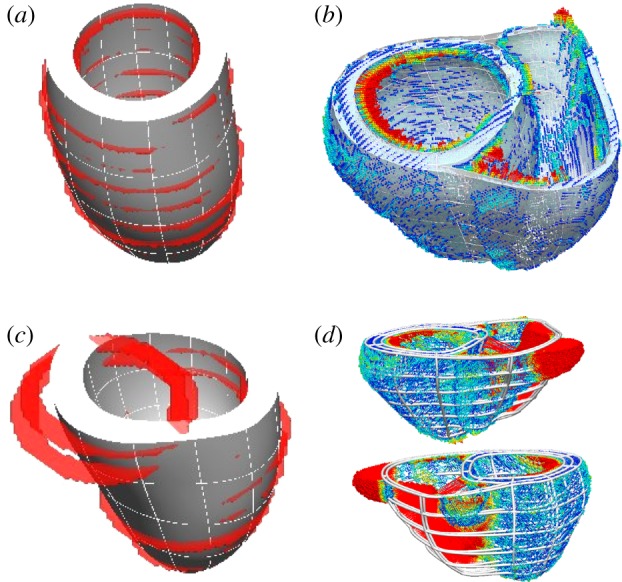
Illustration of accuracy results in the best (*a,b*) and worst (*c,d*) case of each cohort, with average errors of 0.62, 0.74, 4.2 and 3.1 mm for (*a*–*d*), respectively. (*a,c*) illustrate the original data (red isosurface) and final mesh (white), and (*b*,*d*) show the geometrical fitting error colour coded from blue (0 mm) to red (1 mm or more). Large fitting error for the LV in (*c*) is owing to a heavy slice shift. The RV region has the biggest errors in case of the BiV in (*d*).

All 255 meshes were fully automatically fitted to the segmented anatomy with good performance: LV meshes had a median error and quality of 1.18 mm and 0.37, respectively, and 1.62 mm and 0.37 for BiV meshes (see figure 10 for a complete description of the distribution of errors and quality). The best and worst cases in each cohort are shown in figure 11. Defining success as a combination of an average error smaller than the voxel diagonal size and mesh quality above the empirical threshold of 0.33 [22], 87% and 84% of LV and BiV cases, respectively, were successfully personalized.

### 3.6. Computational cost

Computational cost depends mainly on the resolution of the input image, and on the number of elements of the computational mesh. Current implementation (running on a conventional desktop machine) takes between 5 and 25 min to personalize each anatomical case.

## 4. Discussion

The automatic method for personalization of cardiac ventricular meshes is versatile (able to work with different species and disease conditions) and robust (able to work with sparse datasets, and obtaining fully automatic results fulfilling accuracy and quality requirements in 87% of cases). This tool is now available for the scientific community.

The computational meshes built using the proposed method are smooth, owing to the small number of elements and use of high-order interpolation, and remove the sometimes undesired anatomical detail in the heart (see removal of trabecular anatomy in figure 4, and note that these anatomical structures can play a significant role in electrophysiological phenomena [29,30]).

The geometrical smoothing also acts as a regularization that alleviates shape bias introduced by segmentation errors or by the presence of slice shift common in short axis acquisitions (figure 11). Another benefit of the use of smoothing meshes is the relaxation of requirements in accuracy for the image segmentation step. These characteristics make this meshing solution an attractive choice for the description of anatomy, with the approach having already been successfully applied to reveal shape differences between populations with distinct gestational ages [28].

Another strength is the ability to work with very sparse anatomical data from dynamic cardiac imaging studies, where only a few slices are available (figure 9). An alternative approach is the use of image interpolation [31], where recent advances using patch matching are reporting reasonable results at the apex of the ventricles [32]. It should be noted that the proposed mesh fitting method finds an approximate description of the anatomy and does not guarantee the preservation of the original segmentation contours.

The tool is versatile in order to fit a wide range of user requirements, especially for the compromise between fitting accuracy and simulation stability governed by the input parameter LoD. A small LoD is suggested for cases with a limited amount of data, such as dynamic imaging studies with only a few slices or for cases with segmentation errors. Larger values of LoD can be adopted to maximize the anatomical accuracy if there is no need for simulation stability (e.g. shape analysis).

To our knowledge, this is the first solution proposed for the automatic construction of high-order interpolation cardiac meshes from a binary image. Our described methods can be seamlessly integrated with any image segmentation solution that produces a binary mask of the ventricular anatomy, and it is thus complementary with recent advances in automatic cardiac segmentation [19,20,26,27] (note that shape models used for segmentation can also produce a computational mesh, but, to date, only models with linear tetrahedral elements have been employed [19,26,27]). Our solution can also be complemented with methods to align microscopic anatomical data [29], where image registration techniques are also employed to geometrically align myocardial domains (for example, the large deformation diffeomorphic mapping [33]).

**Figure 12.**
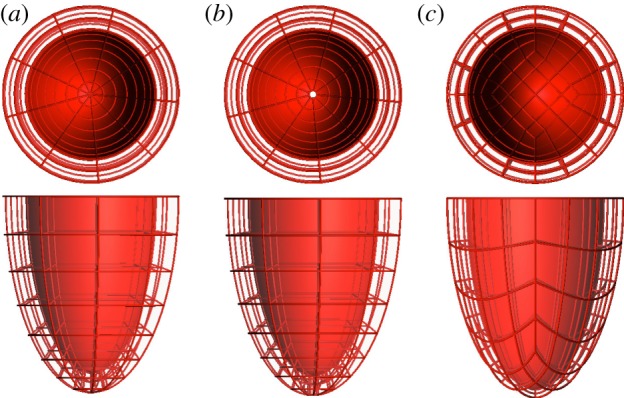
Three mesh template topologies for an LV using structured elements with high-order interpolation. (*a*) With collapsed elements in the apex; (*b*) with an apical hole and (*c*) with a squared patch of elements at the apex. (Online version in colour.)

The choice of the mesh topology in the template (figure 12*a*) is motivated by a set of requirements for the simulation of mechanics. This simplified structured ventricular topology has been a common choice since early works in the field [34,35]. Removal of the valve basal anatomy in order to have a flat horizontal plane at the base is convenient for the prescription of mechanical boundary conditions [3,6,8], and also for better element quality (because thin and skewed elements will be required to represent valve anatomy). This topology has the benefit of the direct mapping between the local and global material coordinates (longitudinal, circumferential and radial) but at the cost of needing collapsed elements at the LV apex. Note that not all solvers will be able to work with collapsed elements, and some authors have circumvented this issue with an apical hole [8] (figure 12*b*). An alternative topology uses a squared patch of elements at the apex with an aspect ratio bounded under grid refinement [36] (figure 12*c*), but at the cost of missing the direct mapping between local and global coordinates.

The main limitation of our choice of a structured BiV topology is the RV apex: the line of insertion of the RV into the LV has a squared shape (figure 2), whereas the anatomy shows that the RV apex is approximately an ellipse [37], as discussed in [22]. As a consequence, the points where the RV attaches to the LV are anatomically incorrect near the RV apex in all meshes with this topology.

Mesh requirements for simulation of electrophysiology, blood flow or perfusion (vasculature) can be very different, with specific requirements for the level of anatomical and physiological detail. The methodology proposed here is aimed at mechanical simulations, with application also including continuum approaches for perfusion modelling [38]. The definition of minimum information required to personalize a mesh will depend on the anatomical accuracy needed for the posterior simulation or analysis. The solution proposed here is versatile to adapt to any imaging modality input from which anatomical information is available to reconstruct the three-dimensional shape (a single short axis slice is typically insufficient).

Proposed mesh personalization solution does not guarantee an exact anatomical correspondence between different cases. Further work is thus required to improve this correspondence, and one solution is the inclusion of fiducial anatomical markers, such as the insertion of the RV into the LV or hinges of valve planes [39]. Another limitation of the study is that results on method robustness are dependent on the thresholds set for accuracy and quality, and these may change depending on application requirements. Specifically, mesh quality threshold in this work is based on an empirical study with specific cardiac mesh and simulation conditions [22] and its generalization has not been analysed.

This service can easily be generalized and extended to other anatomical structures (such as the atria or entire heart [16]) and/or topological requirements (such as the different topologies illustrated in figure 12 or the inclusion of the valve planes). The process will require the definition of a suitable template and an algorithm for initial alignment in order to fall into the capture range of the image registration step. The deployment of this personalization service as part of the VPH-Share cloud infrastructure (http://www.vph-share.eu/) aims to be part of a toolkit for the scientific community to accelerate the clinical translation and provide a research and diagnostic paradigm based on biomedical modelling and simulation of cardiac physiology.
